# Poly(-β-hydroxybutyrate) (PHB) depolymerase PHAZ_*Pen*_ from *Penicillium expansum*: purification, characterization and kinetic studies

**DOI:** 10.1007/s13205-015-0287-4

**Published:** 2015-03-24

**Authors:** Vaishnavi Gowda U. S., Srividya Shivakumar

**Affiliations:** Department of Microbiology, Centre for Post Graduate Studies, Jain University, 18/3, 9th Main, Jayanagar 3rd Block, Bangalore, 560011 India

**Keywords:** PHB depolymerase, *P. expansum*, Production, Purification, Characterization, Biochemical properties, Kinetics

## Abstract

Very few studies have been dedicated to R-hydroxyacids (R-HA) production using extracellular polyhydroxyalkanoate depolymerases (ePhaZs). *Penicillium expansum* produced maximum extracellular polyhydroxybutyrate depolymerase (~6 U/mL) by 72 h when grown in mineral salt medium containing 0.2 % w/v PHB, pH 5.0, at 30 °C and 200 rpm shaking conditions. Partial purification of the extracellular poly(-β-hydroxybutyrate) depolymerase PHAZ_*Pen*_ from *P. expansum* by two steps using ammonium sulphate (80 % saturation) and affinity chromatography using concanavalin A yielded 22.76-fold purity and 43.15 % recovery of protein. The enzyme composed of a single polypeptide chain of apparent molecular mass of 20 kDa, as determined by SDS-PAGE, stained positive for glycoprotein by periodic–schiff base (PAS) staining. Optimum enzyme activity was detected between pH 4.0 and 6.0 at 45–50 °C with pH 5.0 and 50 °C supporting maximum activity. The enzyme was stable between pH 4.0 and 6.0 at 55 °C for 1 h with a residual activity of almost 70–80 %. The enzyme was completely inhibited by 1 mM DTT/1 mM HgCl_2_ and *N*-ethylmaleimide (10 mM) indicating the importance of essential disulphide bonds (cystine residues) and tyrosine for enzyme activity or probably for maintaining the native enzyme structure. Among the various divalent and trivalent metal ions, mercuric chloride, ferric citrate and ferrous sulphate inhibited enzyme activity. The enzyme showed substrate specificity towards only PHB and poly(3-hydroxybutyrate-*co*-3-hydroxyvalerate) and no other lipid or other *p*-nitrophenyl fatty acids or with polycaprolactone, showing that it was a true depolymerase and not any lipase or cutinase. Preliminary investigation revealed *β*-hydroxybutyrate as the end product of PHB hydrolysis by *P. expansum*, suggesting that the enzyme acted principally as an exo-type hydrolase. The above properties when compared with other fungal PHB depolymerases reported till date suggest the distinct nature of the PHB depolymerase of *P. expansum*.

## Introduction

The growing awareness of the importance of chirality in conjunction with biological activity has led to an increasing demand for efficient methods for the industrial synthesis of enantiomerically pure compounds. Polyhydroxyalkanoates (PHAs) are a family of polyesters consisting of over 140 chiral R-hydroxycarboxylic acids (R-HAs), representing a promising source for obtaining chiral chemicals from renewable carbon sources. Since R-hydroxyacids (R-HAs) contain a chiral centre and two easily modified functional groups (–OH and –COOH), they are valuable synthons, i.e. they may serve as starting materials for the synthesis of fine chemicals such as antibiotics, vitamins, flavours, fragrances and pheromones (Seebach et al. 2001; Calabia and Tokiwa 2006; Chiba and Nakai 1985; Ohashi and Hasegawa 1992).

Although some R-HAs have been produced for some time and certain knowledge of the production processes has been gained, large-scale production has not yet been possible (Ren et al. 2010). Currently, methods for production of R-HAs include chemical synthesis, biocatalysis, chemical or enzymatic degradation of biologically synthesized PHAs, in vivo depolymerization of PHAs and biotechnological production by metabolic pathway engineering (Chen and Wu 2005; Ren et al. 2010). Direct chemical synthesis of R-HAs has many limitations such as the requirement of pure substrate and expensive catalysts (Ren et al. 2005, 2010). Lee et al. (2000) reported an efficient method for the preparation of R-3HB by acidic alcoholysis of PHB. De Roo et al. (2002) produced the chiral medium chain length (mcl) (R)-3-hydroxycarboxylic acids via hydrolytic degradation of PHAs synthesized by *Pseudomonas putida*.

A method for producing R-3HB and (R)-3-hydroxyvaleric acid (R-3HV) from PHB and poly[(R)-3-hydroxybutyrate-*co*-(R)-3-hydroxyvalerate] (PHBV) by chemical degradation has been reported (Seebach et al. 1993). In vivo depolymerization of PHA requires the biomass with accumulated PHA to be harvested from the fermentation broth first, and then PHA degradation is enhanced by modifying the pH or temperature (Lee et al. 1999; Ren et al. 2005). Metabolic pathway engineering has been employed to produce R-HAs by over-expression of depolymerase and/or under-expression of synthase or dehydrogenase, but it is still not at a stage of practical use (Chung et al. 2009; Park et al. 2004; Romano et al. 2005; Sandoval et al. 2005).

Till now, many extracellular PHA depolymerases (ePhaZs) have been identified and characterized (Jendrossek and Handrick 2002; Kim et al. 2007). The ePhaZs partially degrade crystallized or denatured PHA, and the degradation products are typically R-HA monomers and/or dimers (Jaegar et al. 1995; Jendrossek and Handrick 2002). ePhaZs have been mainly used for surface modification (Numata et al. 2008), while very few studies have been dedicated to R-HA production using ePhaZs. It has been reported that thermophilic *Streptomyces* sp. MG can hydrolyse purified PHB to R-3HB (Calabia and Tokiwa 2006). The occurrence of extracellular depolymerases is widespread amongst microorganisms as compared to that of intracellular depolymerases (Lee et al. 1999). The PHB hydrolysis can be carried out by incubating the polymer with the purified enzyme.

Before attempting to understand the mechanism of an enzyme’s activity, the enzyme must be purified and isolated to the point that no other enzymes can be detected (Deutscher 1990). Therefore, the purification and the isolation of an enzyme is a very important step, which must be designed very carefully and many factors such as pH, temperature, metal ions, substrate specificity and end products must be considered. The purification process is considered to be successful when the ratio of enzyme activity to the total protein is increased to the limit. For this reason, the enzyme activity and the amount of protein must be determined at every step of the procedure. The risk of failure of this process of isolation and purification, which results in isolating an inactivated enzyme, is big because enzymes are fragile and proteins can denature very easily. Hence, a purification strategy, with minimum steps exploiting some of the properties of the enzyme, which is fast and results in an active and effective isolated enzyme is highly desirable (Panagiotidou et al. 2014). Most of the fungal depolymerases are glycosylated, and hence can be concentrated by ammonium sulphate precipitation and purified in one step using affinity column with concanavalin agarose as the affinity matrix yielding high purification fold and recovery.

The present paper describes a strategic and simple two-step purification process of PHB depolymerase from *P. expansum* yielding appreciably high purification fold and recovery. The exceptional properties of this enzyme and its capability of producing *β*-hydroxybutyrate as the end product of hydrolysis, as revealed by preliminary investigations, are emphasized and compared with those of reported bacterial and fungal depolymerases.

## Materials and methods

All the experiments as described below were repeated at least twice in triplicate.

### Materials

PHB was obtained as a kind gift from Biomer Inc., Germany. The molecular weight of PHB was 470,000 g/mol. All experiments were performed using PHB powder. Polycaprolactone [PCL; MWavg, 42,500] and other substrates were purchased from Aldrich Chemical Co. Suspensions of PCL were prepared as described by Jaegar et al. (1995). All the inhibitors were purchased from Sigma (St. Louis, USA). *p*-nitrophenyl alkanoates-*para*-nitrophenyl acetate (PNPA), *para*-nitrophenyl butyrate (PNPB) and *para*-nitrophenyl decanoate (PNPD) were procured from HiMedia, Bombay. All other reagents were of analytical grade.

### Isolation of a short chain length (SCL-PHA) degrading fungus

An SCL-PHA degrading filamentous fungus from a wastewater sample was isolated by pure culturing a colony with high depolymerase activity among fungi grown on a mineral salt agar medium containing PHB as the sole carbon source (Iyer et al. 2002).

### Production of the PHB depolymerase from the isolate

The isolate was cultivated in modified Bushnell Haas (BHM) agar medium at 30 °C for 3–4 days. The spore suspension was placed into mineral PHB media (0.2 %, w/v PHB, 0.7 g K_2_HPO_4_, 0.7 g KH_2_PO_4_, 0.7 g MgSO_4_, 1.0 g NH_4_Cl, 1.0 g NaNO_3_, 5 mg NaCl, 2 mg FeSO_4_, 7 mg ZnSO_4_ in 1L of D.W.) and cultivated at 30 °C ± 2 for 3 days. The culture supernatant was filtered using Whatman filter paper no. 1 and used as enzyme source (Iyer et al. 2002).

### Protein estimation

Protein concentrations were measured by Bradford’s method (1976) using bovine serum albumin as the standard.

### Enzyme assay

The PHB depolymerase assay was carried out in a 3.0 mL system by adding the enzyme to 100 mM sodium citrate buffer (pH 5.0) containing 300 µg of PHB at 55 °C for 1 h. The activity was stopped by adding 0.1 mL of 1 N HCl. The decrease in the turbidity was measured at 600 nm using a colorimeter. One unit of PHB depolymerase is defined as the amount of enzyme required to decrease the A_600_ nm by 1.0 per hour (Scherer 1996).

### Purification of PHB depolymerase

The extracellular PHB depolymerase from the culture filtrate of the fungus was purified by (NH_4_)_2_SO_4_ precipitation (80 % saturation), followed by affinity chromatography using concanavalin A. The fractions collected were assayed for enzyme activity and protein content by the method mentioned above (Srividya et al. 2011; Srividya 2013). Pre-packed concanavalin A (Con A)-agarose affinity matrix (Bangalore Genei Pvt. Ltd., Bengaluru, India) was used to purify the glycoproteins and the entire procedure was carried out at 4 °C. A column (10 mL) pre-packed with 0.5 mL bed volume of Con A agarose was washed with three bed volumes of equilibration buffer (10 mM sodium acetate buffer, pH 5.0), at a flow rate of 1 mL/min. A total of 2.6 mg precipitated ammonium sulphate and dialysed protein fraction (6.5 mL) were loaded on to the column under gravity. Flow through was collected in 10 mL sterile vials and the column was extensively washed with binding buffer till the absorbance of proteins became zero at 280 nm. For the elution of Con A-binding proteins, the column was washed with elution buffer [sodium actetate buffer (pH 5.0) containing 0.5 M Methyl- d’- mannopyranoside]. The eluted proteins were dialysed against 1 mM sodium acetate buffer (pH 5.0) for 16 h followed by 50 % glycerol and left overnight at 4 °C. The proteins were analysed by SDS-PAGE.

### Molecular weight determination

Electrophoresis (10 %, w/v SDS-PAGE) was carried out to measure the molecular weight according to LaemmLi (1970).

### Glycoprotein staining by periodic acid–Schiff (PAS) method

Carbohydrate staining of glycoprotein in SDS-PAGE gel was carried out with fuchsin-sulphate after periodate oxidation (PAS) according to Zacharius et al. (1969).

### The effects of temperature and pH on the enzyme activity

The temperature optima of PHB depolymerase was obtained by measuring enzyme activity at different temperatures in the range of 25–60 °C at optimum pH. The stability of the enzyme at varied temperatures was measured by incubating the enzyme (1 μg) in standard reaction solution without PHB for 90 min at 25–60 °C. The effect of pH on enzyme activity was determined by using buffers of pH 3.0–4.0 (0.1 M sodium acetate), pH 5.0–6.0 (0.1 M sodium citrate) and pH 7.0–8.0 (0.1 M phosphate). The optimum pH for enzyme activity was determined by measuring the activity in each of the buffers containing PHB (300 μg) and enzyme (1 μg). The pH stability of the enzyme was determined using buffers containing enzyme without substrate incubated at 25 °C for 2 h, and then controlled to have the optimum pH to measure the residual activity of the enzyme (Iyer et al. 2002).

### Determination of *K*_m_ and *V*_max_

A PHB stock solution (3 mg/mL) was used to prepare varying PHB (substrate) concentrations from 200 to 2000 μg/mL, making up the volume to 2 mL with buffer (optimal assay pH). Four such sets of varying concentrations of substrate were prepared, one for each range and the blank. 1 mL of enzyme extract containing 1 μg enzyme was added to each of the tubes of the respective set. The tubes were incubated at 50 °C for 1 h. The reaction was stopped by adding 0.1 mL of 1 N HCl and the absorbance was read at 600 nm against a blank for each tube. The apparent *K*_m_ and *V*_max_ values of PhaZ_*Pen*_ for PHB hydrolysis were calculated by non-linear hyperbolic regression, using the starting values obtained by linear regression fitting of a Hanes–Woolf plot (Wilkinson 1961; Duggleby 1981) with the Hyper32 software (freely available at http://homepage.ntlworld.com/john.easterby/hyper32.htmL). These parameters were calculated using the turbidimetric activity assay with PHB, the natural substrate of PhaZ_*Pen*_, and considering a PHB weight average molecular mass (*M*_W_) of 470 kDa, provided by the manufacturer.

### The effect of metal ions

To study the effect of metal ions on PHB depolymerase activity, 0.08 mL of each of the metal ion solutions (1 mM) was mixed with the enzyme–buffer system (4 mL containing 1 μg enzyme and 300 μg PHB) along with the blank and incubated at 50 °C (optimum assay temperature) for 1 h. The reaction was stopped by adding 0.1 mL of 1 N HCl and the absorbance was read at 600 nm. The metal ions used were calcium chloride (CaCl_2_), magnesium sulphate (MgSO_4_), ferric citrate (C_6_H_5_FeO_7_)·xH_2_O ferric chloride (FeCl_3_), cobalt chloride (CoCl_2_), cadmium nitrate [Cd(NO_3_)_2_] and manganese acetate [Mn(CH_3_COO)_2_] (Han and Kim 2002).

### Substrate specificity

The standard assay under optimal conditions was performed for the enzyme extract using P (HB-co-HV) (5 %) and PCL (5 %) as the substrate instead of PHB. The absorbance was read at 600 nm against a blank (Iyer et al. 2002).

### Esterase activity

Esterase activity was assayed in 2 mL of 100 mM sodium citrate buffer, pH 6.0, using *p*-nitrophenyl alkanoates-*para*-nitrophenyl acetate (PNPA), *para*-nitrophenyl butyrate (PNPB) and *para*-nitrophenyl decanoate (PNPD) incubated at 37 °C for 10 min. The reaction mixtures contained 50 µL of a 10 mM solution of the substrates in ethanol and 10 µL (containing 1 μg) of the enzyme solution. The reaction mixture without enzyme was taken as control. The reaction was stopped using 0.1 mL of 1 M Na_2_CO_3_ to enhance the colour of released PNP. One unit of esterase activity was defined as the amount of protein required to produce 1 μmol of PNP from the substrate per minute (Kim et al. 2002).

### Effect of inhibitors

The inhibitory effect of various chemical reagents, diazo-dl-norleucine methyl ester (DAN), *N*-*p*-tosyl-l-lysinechloromethyl ketone (TLCK), *N*-ethylmaleimide (NEM), *N*-acetylimidazole (NAI), iodoacetate (IA), dithiothreitol (DTT), phenylmethylsulphonyl fluoride (PMSF), EDTA, SDS and urea, on enzyme activity was measured as follows: the reaction mixture (1.97 mL) containing 1 mL (1 μg) of the enzyme solution, reagent (10 mM) and sodium citrate buffer (100 mM, pH 6.0) was initially pre-incubated for 1 h at 37 °C. The enzymatic reaction was subsequently started by adding 2 mL of the PHB (300 μg) substrate. (Kim et al. 2002, 2004).

## Results and discussion

### Microorganism: identification and characterization

The PHB degrading fungal colonies were rapidly growing, fasciculate to synnematal; conidial mass dull green; exudates and soluble pigment brown. Microscopic features included: conidiophores stipes smooth walled, 200–500 μm long; penicillin terverticillate, metulae 12–18 μm long; philalides closely packed, flask shaped, tapering into a short, narrow neck, 8–11 μm long; conidia ellipsoidal, smooth walled, 3.0–3.5 μm long. Based on these characteristics, the strain was identified as *Penicillium expansum* (Fungal Identification Service, Agharkar Research Institute, Pune, India) by morphotaxonomy. *Penicillium expansum* produced maximum PHB depolymerase (~6U/mL) by 48 h when grown in BHM containing 0.2 %, w/v PHB, pH 5.0, at 30 °C.

The enzyme production depends on the culture time and the temperature at which the microorganism grows. According to the literature, the maximum quantity of PHB is being produced in the stationary phase at 30 °C (Han et al. 1998; Han and Kim 2002).

### Partial purification and characterization

**Fig. 1:**
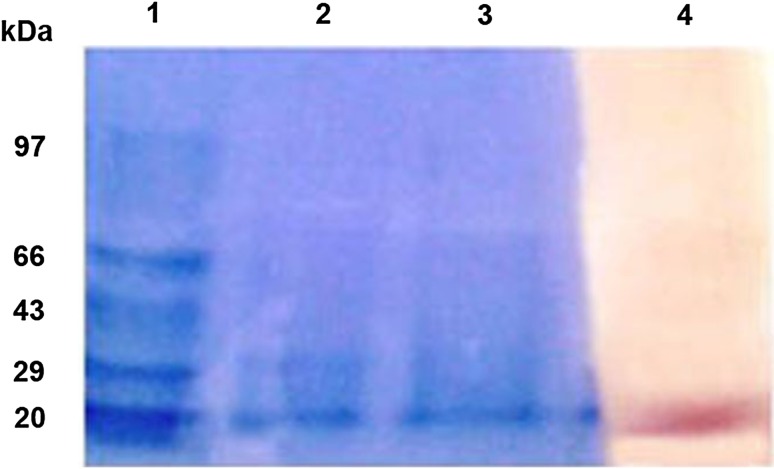
SDS denatured PAGE-standard molecular weight marker [Coomassie stained (*Lane 1*), purified PHB depolymerase by concanavalin A (*Lane 2*, *Lane 3*) and glycoprotein staining of the purified PHB depolymerase of *P. expansum* by PAS method (*Lane 4*)]

Partial purification of the extracellular poly(-β-hydroxybutyrate) (PHB) PHAZ_*Pen*_ from *P. expansum* using ammonium sulphate (80 % saturation) followed by affinity chromatography using concanavalin A yielded 22.76-fold purity with 43.15 % recovery of protein (Table 1). The enzyme was composed of a single polypeptide chain of apparent molecular weight of 20 kDa, as determined by SDS-PAGE (Fig. 1, lanes 2, 3). The enzyme also stained positive for glycoprotein by PAS method (Fig. 1, lane 4).

**Table 1:** Purification of *P. expansum* PHB depolymerase

Sample	Activity (U/mL)	Total activity (units)	Protein (mg/mL)	Total protein (mg)	Specific activity (U/mg)	Purification fold	% Recovery
Crude (500)	4	2000	0.05	0.025	80	1	100
NH_4_(SO_4_) cut 80 % saturation (6.5 mL)	336	2184	0.4	2.6	840	10.5	92.38
Affinity chromatography using concanavalin A agarose (10 mL)	102	1020	0.056	0.56	1821	22.76	43.15

Han et al. (1998) isolated extracellular PHB depolymerase from *Penicillium pinophilum* (ATCC 9644) using three chromatography columns with purification fold 2.1. Han and Kim (2002), who used another fungus, *Penicillium simplicissimum* LAR13, and one chromatography column, increased the enzyme activity 2.1-fold. Brucato and Wong (1991) purified extracellular PHB depolymerase from *Penicillium funiculosum* applying hydrophobic chromatography with purification fold 4.5.

In this work, application of a simple two-step purification method, the precipitation with ammonium sulphate followed by affinity chromatography, resulted in a purified enzyme with activity 22.76-fold that was much higher compared to that obtained from the literature using muti-step purification methods. The total protein from the culture filtrate was concentrated by ammonium sulphate precipitation and the PHB depolymerase was isolated from the contaminating proteins (as evidenced by the drastic decrease in the protein content) by affinity chromatography using Con A agarose which specifically bound PHB depolymerase of glycoproteinic nature, yielding a high purification fold of PHB depolymerase. Earlier also, we have reported such high purification fold and recovery with a similar strategy for *Fusarium solani* Thom and *P. citrinum* S2 PHB depolymerases (Srividya et al. 2011; Srividya 2013) and suggest this two-step simple method for purification of fungal PHB depolymerases to get high purification fold and recovery for all fungal PHB depolymerases.

The molecular weight of *P. expansum* PHB depolymerase determined here is in agreement with that of the PHB depolymerase obtained from many fungal (Brucato and Wong 1991; Iyer et al. 2002; Kim et al. 2002; Han et al. 1998) and bacterial PHB depolymerase (Jeong 1996; Sadocco et al. 1997; Nakayama et al. 1985; Kita et al. 1995), all of which showed single polypeptides of varied molecular weights. The PHB depolymerase of *A. faecalis* AE122 and *F. solani* Thom are the only exceptions with depolymerases reported with unusually high apparent *M*_r_ of 96 KDa and 85 KDa, respectively (Kita et al. 1995; Srividya 2013). In contrast, the PHB depolymerases of *P. citrinum* S2 exhibited three polypeptides with 66, 43 and 20 KDa, respectively (Srividya et al. 2011).

The enzyme stained positive for glycoprotein by PAS staining. Carbohydrates were not detected in the PHB depolymerase either from *A. fumigatus* or from *A. saperdae* (Scherer 1996; Sadocco et al. 1997). However, the bacterial PHB depolymerases from *Pseudomonas lemoignei* (Nakayama et al. 1985) and fungal PHB depolymerase from *P. funiculosum* (Brucato and Wong 1991), *P. simplicissimum* (Kim et al. 2002), *A. fumigatus* Pdf1 (Iyer et al. 2002), *F. solani* Thom (Srividya 2013) and *P. citrinum* S2 (Srividya et al. 2011) were glycosylated. The composition and function of the carbohydrate moiety of the glycosylated PHB depolymerase and other proteins studied so far are unknown. A functional role of the carbohydrate moiety in binding the substrate of cellulases has been proposed. However, glycosylation is not essential for activity, but may prove the resistance of the exoenzyme to elevated temperature and/or hydrolytic cleavage by proteases of competing microorganisms (Briese et al. 1992).

### Biochemical properties and kinetics of purified PHB depolymerase

**Fig. 2:**
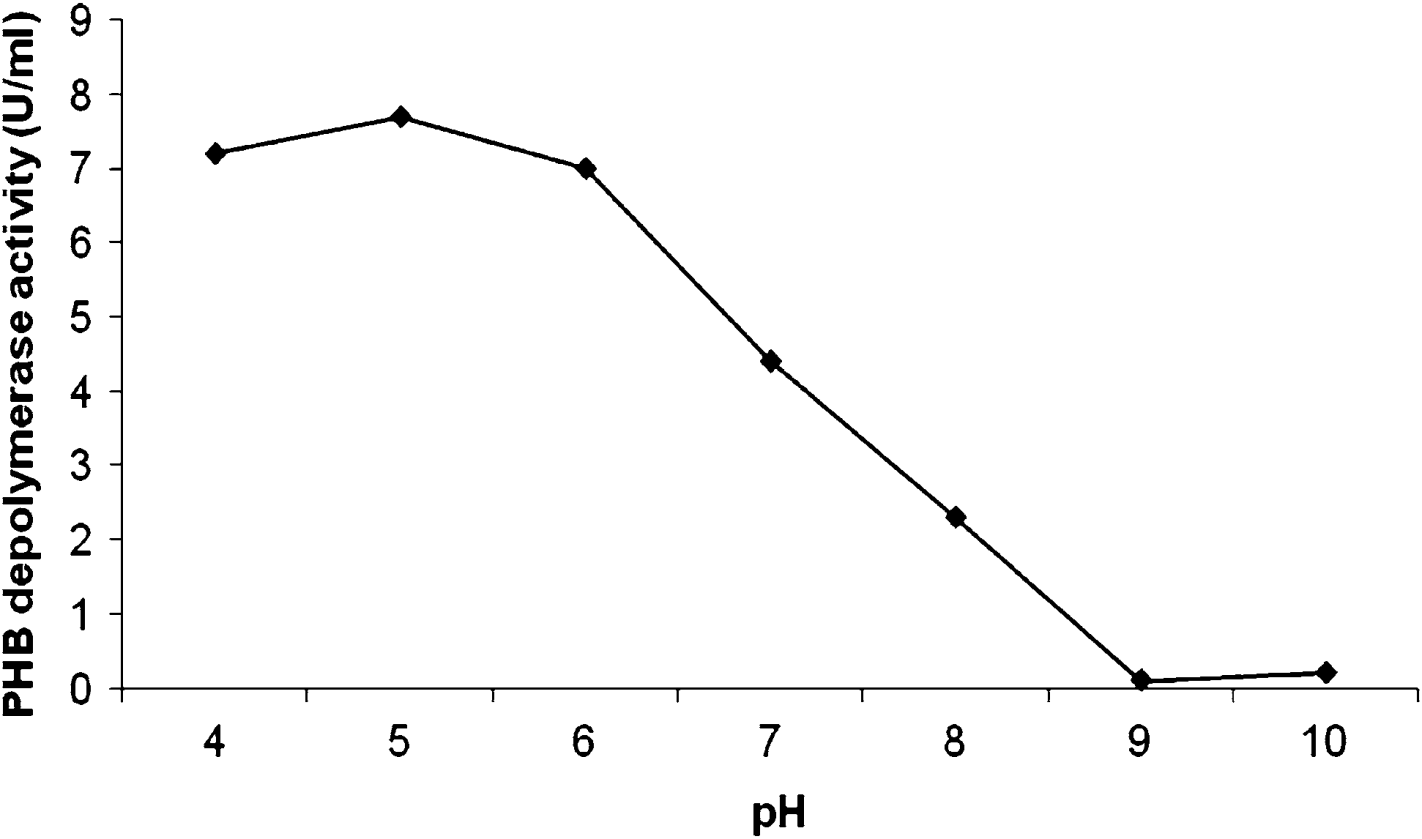
pH optima of *P. expansum* PHB depolymerase activity

**Fig. 3:**
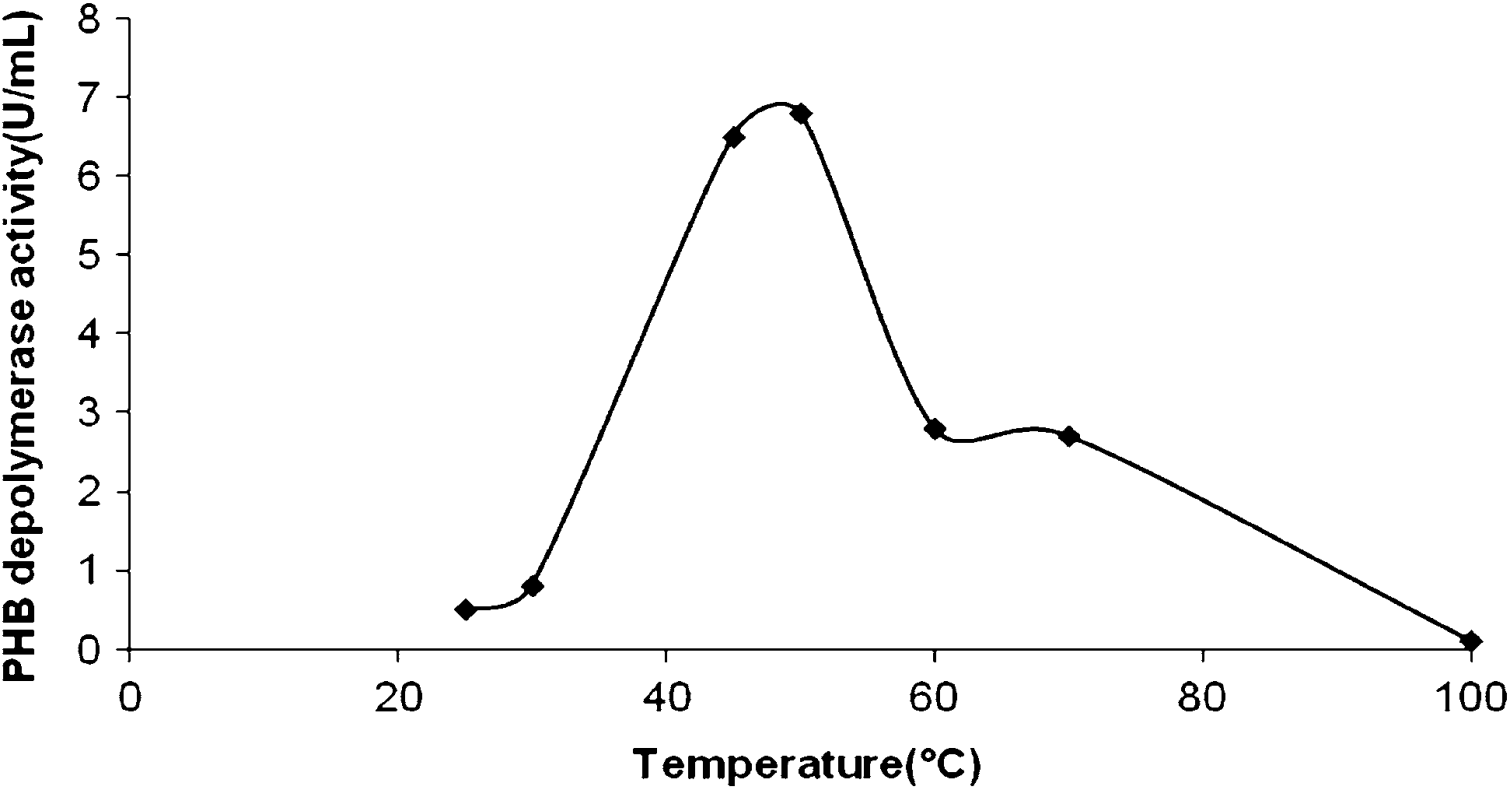
Temperature optima of *P. expansum* PHB depolymerase activity

The optimum enzyme activity for *P. expansum* PHB depolymerase was detected between pH 4.0 and 6.0, the highest being at pH 5.0 (Fig. 2) and at 50 °C (Fig. 3). The enzyme was stable at pH 4.0, 5.0 and 6.0 and 55 °C for 1 h with a residual activity of almost 70–80 %.

**Fig. 4:**
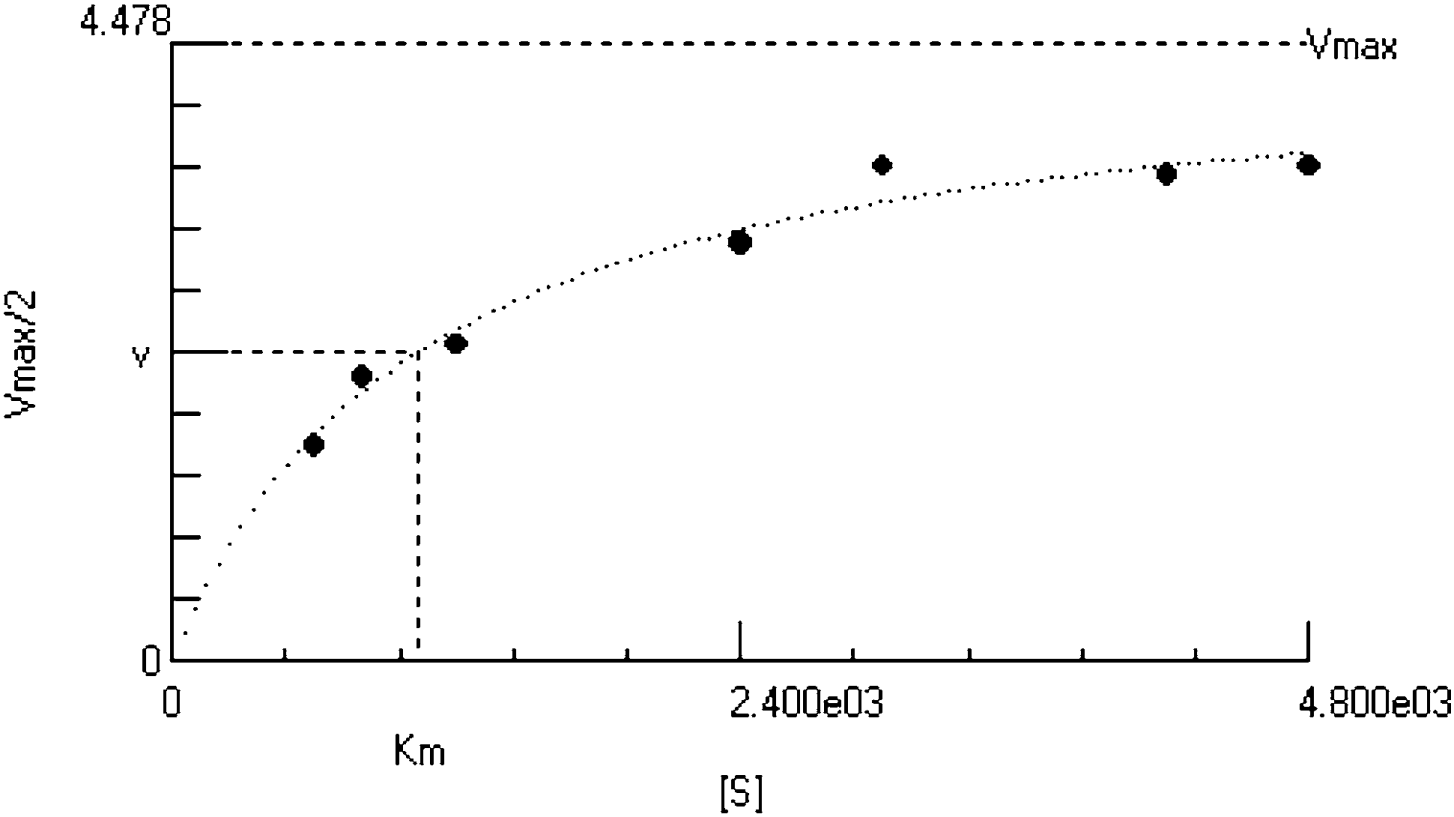
Determination of apparent *K*_m_ and *V*_max_ of *P. expansum* PHB depolymerase by hyperbolic regression of Hanes–Woolf plot

Furthermore, the kinetic parameters of PhaZ_*Pen*_ for PHB hydrolysis were also determined. The apparent *K*_m_ and *V*_max_ values were 1.04 μg/mL and 4.5 μg/min, respectively (Fig. 4). The kinetic parameters of recombinant PHAZ_*sa*_ of *Streptomyces ascomycinicus* for PHB hydrolysis determined by the nonlinear regression gave apparent *K*_m_ and *V*_max_ values of 0.61 ± 0.11 μm and 9796.8 ± 186.8 U/mg of enzyme, respectively (García-Hidalgo et al. 2013). The apparent *K*_m_ value of the purified enzyme of *Thermus* *thermophilus* HB8 for PHB was 53 μg/mL. (Papaneophytou et al. 2009). The apparent *K*_m_ and *V*_max_ values of *F. solani* Thom were found to be 100 μg/mL and 50 μg/min, respectively (Srividya 2013). The apparent *K*_m_ and *V*_max_ values of *P. citrinum* S2 PHB depolymerase was found to be 1250 μg/mL and 12.5 μg/min, respectively (Srividya et al. 2011). The kinetic parameters thus show that the PHB depolymerase of *P. expansum* seems to have a very high affinity for PHB as compared to the other depolymerases reported.

The enzyme was thermally as stable as other PHB depolymerases reported (Scherer 1996; Nojima et al. 1996; Brucato and Wong 1991; Srividya 2013; Srividya et al. 2011). The PHB depolymerase produced by PHB decomposing organisms is reported to have high activity and stability at high temperature (Mergaert et al. 1995; Han and Kim 2002).

Among the various divalent and trivalent metal ions HgCl_2_, Ferric citrate and ferrous sulphate inhibited enzyme activity (Table 2). PHB depolymerases show varied response to metal ions (Srividya et al. 2011; Srividya 2013).

**Table 2:** Effect of different metal ions and inhibitors on *P. expansum* PHB depolymerase

Reagent	Relative activity (%)
Control	100
Metal ions (1 mM)	
HgCl_2_	18
CaCl_2_	91
CuCl_2_	59
FeCl_3_	87
MgCl_2_	89
Cd(NO_3_)_2_	87
[Mn(CH_3_COO)_2_]	86
Fe_2_SO_4_	16
(C_6_H_5_FeO_7_)·xH_2_O	03
Inhibitors (10 mM)	
DAN	53
TLCK	54
NEM	0
NaI	40
IA	35
DTT	0
PMSF	41
EDTA	59
SDS	16
Urea	46
NAM	37

Inhibitors are indicative of the various functional groups present in the active site of an enzyme. The effect of inhibitors on the activity of the enzyme was investigated to identify the residues at active sites in the PHB depolymerase of *P. expansum*. The enzyme was completely inhibited by 1 mM DTT/1 mM HgCl_2_ and *N*-ethylmaleimide (10 mM) indicating the importance of essential disulphide bonds (cystine residues) and tyrosine for enzyme activity or probably for maintaining the native enzyme structure (Table 2). In contrast to the inhibitor profile of *P. expansum* PHB depoymerase, *P. simplicissimum* LAR 13 PHB depolymerase was not affected by *N*-acetylimidazole (NAI) (Han and Kim 2002). Phenylmethylsulphonyl fluoride (PMSF) is known to be an inhibitor of serine residues (Brucato and Wong 1991; Nakayama et al. 1985; Catherine et al. 1996). The PHB depolymerase of *A. saperdae* (Sadocco et al. 1997) was partially inactivated by 10 mM PMSF, and that of *P. lemoignei* (Nakayama et al. 1985) and *Agrobacterium* sp. (Nojima et al. 1996) was completely inhibited by 1 mM PMSF. However, the PHB depolymerase of *P. simplicissimum* LAR 13 showed 58 % activity in the presence of 10 mM PMSF (Han and Kim 2002). The inhibitor studies clearly show the distinct nature of *P. expansum* PHB depolymerase active site as compared to the PHB depolymerases reported till date.

**Fig. 5:**
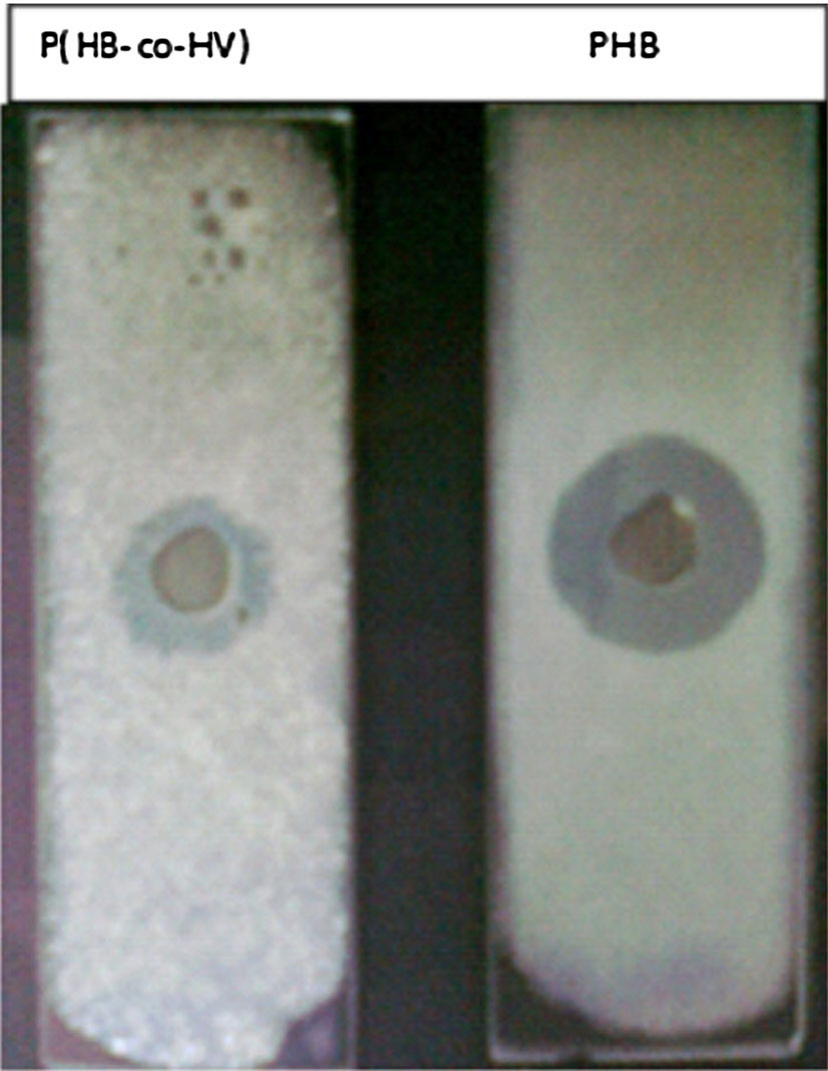
Substrate specificity of *P. expansum* PHB depolymerase with PHB and P(HB-co-HV)

The enzyme showed substrate specificity towards only PHB and P(HB-co-HV) (Fig. 5) and no other lipid or other *para*-nitrophenylalkanoates such as PNPA, PNPD or PNPP, or with polycaprolactone. PNP-acetate and PNP-butyrate were hydrolysed efficiently by the PHB depolymerase of *E. minima* (Kim et al. 2002) and *A. fumigatus* Pdf1 (Iyer et al. 2002). The enzyme activity towards only PHB and P(HB-co-HV) and no other lipid or other *para*-nitrophenylalkanoates such as PNPA, PNPD or PNPP, or with polycaprolactone, shows the true depolymerase nature of the enzyme similar to *P. citrinum* S2 and *F. solani* Thom PHB depolymerases (Srividya et al. 2011; Srividya 2013) lacking any lipase or cutinase activity (Catherine et al. 1996).

**Fig. 6:**
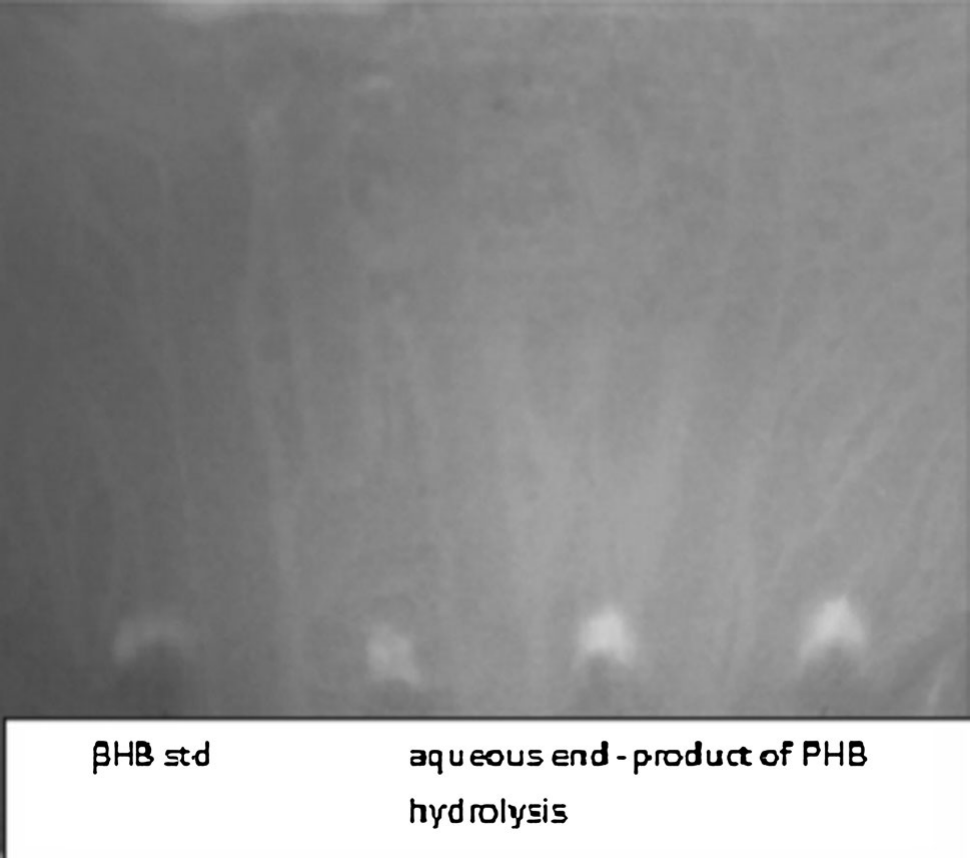
Identification of PHB hydrolysis end products by PHB depolymerase of *P. expansum.* *Lane 1* β-hydroxybutyrate standard, *lanes 2*–*4* end product of PHB depolymerase reaction

Identification of the aqueous end products of PHB depolymerase reaction by paper chromatography revealed *β*-hydroxybutyrate monomer as a major end product of PHB hydrolysis (Fig. 6). The preliminary observation by paper chromatography indicated *β*-hydroxybutyrate monomers as the aqueous end product of PHB hydrolysis by *Penicillium expansum* PHB depolymerase as in the case of *Comamonas* sp. (Chiba and Nakai 1985), *P. Picketti* (Yamada and Mukai 1993), *Thermus* *thermophilus* HB8 (Papaneophytou et al. 2009), *P. citrinum* S2 (Srividya et al. 2011) and *F. solani* Thom (Srividya 2013) as against the depolymerase of *A. faecalis* T1, *A. faecalis* AE122 and *P. lemoignei* which hydrolyse PHB mainly to the dimeric and trimeric ester of hydroxybutyrate (Oda et al. 1997; Kim et al. 2002; Nakayama et al. 1985; Kita et al. 1995). However, high-resolution analysis or HPLC/LC–MS analysis is required to confirm the above observations (García-Hidalgo et al. 2013).

An enantiomerically pure monomer of PHB, d-3-hydroxybutyric acid is an important precursor of 4-acetoxyazetidinone, which is used for making carbapenem antibiotics (Lee et al. 1999). On further investigations, the strain can be exploited for biotechnological applications.

## Conclusion

PHB depolymerase of *Penicillium* *expansum* (PhaZ_*Pen*_) is distinct from other eukaryotic depolymerases in its M*r* and glycosylation and similar to other fungal depolymerases in terms of pH and temperature optima on activity. The enzyme also shows distinct behaviour towards different inhibitors tested which suggests the role of tyrosine and essential disulphide bond (cystine residues) groups in its active site. The present results suggest that PHB depolymerase of *Penicillium* *expansum* (PhaZ_*Pen*_) is an enzyme with distinct characteristics, different from those of other eukaryotic PHB depolymerases reported to date. The simple two-step purification strategy used in this study, with ammonium sulphate precipitation for concentrating the total proteins followed by affinity chromatography exploiting the glycoprotein nature of fungal PHB depolymerases, holds promise in yielding good recovery of highly purified enzyme which can be explored further for in vitro production of the chiral monomer *β*-HB for use as synthons in various pharmaceutical purposes (Chiba and Nakai 1985).
